# On the interplay of borderline personality features, childhood trauma severity, attachment types, and social support

**DOI:** 10.1186/s40479-022-00206-9

**Published:** 2022-12-19

**Authors:** Anna Schulze, Leonie Cloos, Monika Zdravkovic, Stefanie Lis, Annegret Krause-Utz

**Affiliations:** 1 Department of Clinical Psychology, Central Institute of Mental Health, Medical Faculty Mannheim, Heidelberg University, Heidelberg, Germany; 2 Institute of Clinical Psychology, Leiden University, Leiden, The Netherlands; 3 Research Group of Quantitative Psychology and Individual Differences, Faculty of Psychology and Educational Sciences, KU Leuven, Leuven, Belgium; 4 Department of Psychosomatic Medicine and Psychotherapy, Central Institute of Mental Health, Medical Faculty Mannheim, Heidelberg University, Heidelberg, Germany

**Keywords:** Adverse childhood experiences, Borderline personality disorder, Attachment, Perceived social support, Network analysis

## Abstract

**Background:**

Adverse childhood experiences (ACE) have consistently been associated with borderline personality disorder (BPD). Still, it is not yet entirely understood if and how different types of ACE (emotional, physical, sexual abuse, neglect) relate to different BPD subdomains (affective instability, identity disturbance, negative relationships, self-harm). Insecure attachment and lower perceived social support are associated with both ACE and BPD and may therefore contribute to their relationship. No study so far integrated all these variables in one model, while accounting for their mutual influence on each other. We investigated the interplay of BPD subdomains, ACE, attachment, and perceived social support using a graph-theoretical approach.

**Methods:**

An international sample of 1692 participants completed the Childhood Trauma Questionnaire (CTQ), the Borderline Feature Scale from the Personality Assessment Inventory (PAI-BOR), the Adult Attachment Scale (AAS), and Multidimensional Scale of Perceived Social Support (MSPSS) via an online survey. We estimated a partial correlation network including subscales of the CTQ and the PAI-BOR as nodes. We extended the network by including subscales of the AAS and MSPSS as additional nodes.

**Results:**

Emotional abuse was the most central node in both networks and a bridge between other types of ACE and BPD features. All domains of BPD except affective instability were associated with emotional abuse. Identity disturbances was the most central node in the BPD network. The association between ACE and BPD features was partly but not fully explained by attachment and social support.

**Conclusion:**

Our findings suggest that emotional abuse is an important link in the association between ACE and BPD features, also when taking attachment and social support into account. Findings further suggest an outstanding role of identity disturbance, linking emotional abuse to affective instability and being strongly associated with attachment anxiety.

**Supplementary Information:**

The online version contains supplementary material available at 10.1186/s40479-022-00206-9.

## Introduction

Adverse childhood experiences (ACE), such as emotional, physical and sexual abuse, and neglect, can interfere with emotional and social development [1–3]. This is associated with a variety of adverse health outcomes [4, 5], including an increased risk of mental disorders, such as borderline personality disorder (BPD). A recent meta-analysis revealed higher rates of childhood abuse and neglect in individuals with BPD than in healthy controls and other psychiatric groups [6, 7]. Particularly strong associations were reported for emotional abuse [8–10] and sexual abuse [8, 11–15]. When differentiating between specific BPD symptoms, emotional maltreatment (abuse and neglect) was specifically linked to emotional dysregulation in BPD [16]. Sexual abuse significantly predicted behavioural dysregulation [17], suicidal behaviour [11, 18], dissociation, and sexual revictimization [19]. At the same time, ACE are neither a necessary nor sufficient etiological factor for BPD. The disorder develops from a complex interplay of multiple factors, including both vulnerability factors and environmental factors as well as attachment experiences [6, 20, 21]. Central BPD features, such as affective instability, identity disturbance, self-harming impulsivity, and relationship problems, typically onset in adolescence or early adulthood [22].

Attachment insecurity and a lack of perceived social support often co-occur with or follow ACE. Thus, they may partly contribute to the development of BPD features in those with ACE [23–25]. So-called working models of attachment emerge during early interaction with primary caregivers. These working models later serve as a basis for interpersonal relationships [26–28]. They involve expectations and concerns regarding the availability and responsiveness of an attachment figure in times of need, including reactions to separation [29]. Attachment anxiety (i.e., feelings of insecurity related to the availability and interest of others) and attachment avoidance (i.e., dismissal of emotional closeness and dependence) are two main dimensions of insecure attachment [30], which have been linked to ACE [31].

Insecure attachment has been associated with an altered perception of social support, i.e., that others are available when needed [25]. While perceived social support does not necessarily reflect objective support [32], it is an important protective factor in times of distress. It has been shown to have a buffering effect against negative life events [33–36], also in individuals with ACE [37, 38].

In previous research, individuals with insecure attachment perceived ambiguous messages as less supportive than securely attached individuals [39, 40]. Attachment avoidance, in particular, was related to a denial of distress and unwillingness to seek support [41, 42]. Moreover, individuals with insecure attachment were less likely to seek and find support and comfort in their social relationships [36, 43]. The ability to form and maintain trusting relationships is crucial for establishing and relying on social support networks in times of need [43]. This ability seems to be impaired in individuals with BPD.

In fact, a striking clinical characteristic of BPD is that individuals with the disorder feel less socially connected [44], even in experimental conditions designed to signal social inclusion ([44, 45], e.g. [46]). In a study by Lazarus, Southward and Cheavens [47], people with BPD indicated smaller social networks, described more disrupted relationships, and were less satisfied with their perceived support, while no differences in actual closeness of relationships were found [47]. Previous studies revealed strong associations between insecure attachment and the severity of BPD features [23–25]. Individuals with BPD show attachment styles characterized by a longing for intimacy paired with concern about dependency and rejection (i.e., unresolved, preoccupied, and fearful) as well as a negative self-image (see [1, 3, 23, 24, 48–50]). Unintegrated contradictory representations of the self and significant others may contribute to identity diffusion [51]. Maladaptive identity formation is assumed to be a critical part of BPD, which interferes with personality functioning [52]. While their self-image is usually extremely negative, individuals with BPD experience rapid changes in identity and a fragmented sense of self, which can lead to problems in goal-directed behaviour and to difficulties maintaining long-term relationships [52].

All in all, numerous theories and empirical research suggest strong associations between BPD features, ACE, attachment insecurity, and a lack of perceived social support. Associations between these variables may not only be found in a full-blown clinical disorder, but also in people with sub-clinical expressions of BPD.

Notably, these relations may be bidirectional in nature. Together with attachment anxiety, a lack of social support may contribute to the development of BPD features in those with ACE. At the same time, individuals with more severe BPD features may report less perceived social support [47]. In a similar vein, insecure attachment might not only be a risk factor for the development of BPD, but also a consequence of the phenotypical interpersonal impairments in the disorder. Different BPD features and different forms of ACE are usually highly interrelated [53], which hinders a straight-forward investigation of their inter-relations. In the present study, we aimed to get a better understanding of this proposed complex interplay by integrating all variables in one model, while accounting for their mutual influence on each other.

Graph-theoretical analysis, i.e., network analysis, constitutes a state-of-the-art approach to analyze such a complex multivariate interplay of variables. It is a method particularly suited for cross-sectional data and relationships that might be bi-directional or correlational in nature. The resulting networks can visualize the co-occurrence of certain elements (psychopathological symptoms, variables), taking their mutual influence into account [54, 55]. In a graph, this mutual influence is visualized as a set of nodes (elements) and edges (i.e., associations amongst these elements). Each node’s connections can be quantified as an index of centrality, where nodes with more connections have higher centrality indices within the network, accounting for all elements [56]. Therefore, network analyses are particularly helpful for generating hypotheses about causal structures that need to be empirically tested afterwards, even if no specific assumptions can be made beforehand [57, 58]. In clinical research, network analyses also provides a new framework for designing treatments by identifying possible target symptoms [59]. It allows the investigation of complex interplays of symptoms with non-symptom variables and environmental factors, e.g. life events [60]. In the long run, this may not only deepen the understanding of such inter-relations but also help identify variables that connect different clusters of variables. These bridge symptoms may be important targets for interventions.

A growing number of studies has shown that network analysis is a valuable data-driven tool to explore the complexity of disorders, such as BPD [61–63]. Several studies also used a graph-theoretical approach to explore pathways linking ACE to psychopathological symptoms (e.g., [64–66]). To our knowledge, no study so far used a graph-theoretical approach to explore links between BPD features, different types of ACE, attachment anxiety, and perceived social support. Here, we investigated 1) whether different BPD features (affective instability, identity disturbance, self-harming impulsivity, relationship problems) are differentially linked to distinct forms of ACE (emotional abuse, physical abuse, sexual abuse, emotional neglect, physical neglect) and 2) how inter-individual differences in the two dimensions of attachment insecurity (anxiety, avoidance vs. security), and current perceived social support (by family, friends, and a significant other) account for this potential association. In other words, we investigated whether there is a unique association between BPD features and ACE that is not explained by a common association with perceived social support and attachment anxiety.

Based on previous research, we hypothesized that all BPD features are related to all types of ACE, with particularly strong associations for emotional abuse/neglect and sexual abuse. Secondly, we expected that the relationship between BPD and ACE is partly nut not fully explained by perceived social support and attachment anxiety. More specifically, based on the aforementioned literature, we expected that insecure attachment, especially attachment anxiety, partly accounts for the link between BPD features and ACE [24, 25]. We further hypothesized a negative association between attachment anxiety and perceived social support, that is, higher attachment anxiety is related to lower perceived social support.

## Methods

### Participants and procedure

The study was conducted at Leiden University in the Netherlands, after approval by the local Psychology Ethics Committee (CEP19-0307/174). Data collection took place between March 2016 and May 2020. An international sample of *N* = 1682 participants aged between 18 and 65 (*M* = 25.98, *SD* = 8.98) was recruited online. Platforms included international mental health online platforms for people who experienced domestic partner violence during childhood and adulthood (administrators had permitted to post the survey on their homepage), as well as through other social media (Facebook, Instagram, Twitter, etc.) and via the research participation website of Leiden University. Inclusion criteria were age ≥ 18 and having sufficient English proficiency (defined as the ability to “understand the main points of clear standard input on familiar matters regularly encountered in work, school, and leisure”, as checked before and after the survey). Participants were mostly female (*n* = 1215, 72%) and European (*n* = 834, 50%). Almost half of the participants were currently single (*n* = 781, 46%) and finished secondary education (*n* = 745, 44%).

From the initial sample, a subset of *n* = 1102 individuals provided full information about attachment and perceived social support (age 18 – 65, *M* = 26.89, *SD* = 9.44). Again, most participants were female (*n* = 812, 74%), European (*n* = 430, 39%), single (*n* = 470, 43%) and had finished secondary education (*n* = 475, 43%).

Data was collected via an online survey using the software Qualtrics (© 2015, Qualtrics, Provo, UT). Participants could access the survey via a link and a QR code. In the information letter, they were informed about background, aims, potential risks, reimbursement for study participation, and their right to end the survey any time without adverse consequences. A disclaimer was added, highlighting the sensitive nature of the questions: “Please do not participate in this survey if you are in a current crisis or very upset about certain events. Participating in this survey might induce emotional distress (e.g., trigger unpleasant memories, feelings, and thoughts).” To start the survey, participants had to confirm that they met the inclusion criteria and give informed consent, otherwise the survey was automatically terminated. After providing information on demographic variables, participants completed the scales, presented in a randomized order. Upon completion, participants were debriefed and encouraged to contact the principal investigator (AKU), a trained clinical psychologist, in case of discomfort due to the intimate nature of the items. 5 participants contacted the PI. Problems experienced due to the nature of the questionnaire did not require any further intervention. Completion of the survey took approximately 20–30 min. Respondents had the opportunity to participate in a lottery (chance of winning Amazon© vouchers). Psychology students could alternatively gain credits for their participation.

### Measures

#### Borderline personality features

Self-reported core features of BPD were assessed with the Borderline Feature Scale from the Personality Assessment Inventory (PAI-BOR; [67]). Twenty-four items are answered on a 4-point Likert scale (0 – 3; total score range from 0 – 72 with higher scores indicating more severe BPD features). The PAI-BOR has four subscales: affective instability, identity disturbance, negative relationships, and self-harm. The scale has demonstrated high internal consistency, convergent, and concurrent validity, as well as applicability in clinical and non-clinical samples – overall supporting its construct validity [68, 69]. In the present study, internal consistency was α = 0.90.

#### Adverse childhood experiences

We measured different ACE with the Childhood Trauma Questionnaire – Short Form (CTQ; [70]). The retrospective self-report scale consists of 28 Items, answered on a 5-point Likert scale (1 – 5). Three items are designed to capture minimization and denial of problems. Five subscales with five items each assess childhood emotional, physical, and sexual abuse, as well as emotional and physical neglect. Higher scores indicate more severe neglect and/or abuse. The CTQ provides clinical cutoffs that classify into groups of severity [71]. The CTQ showed good psychometric properties [72]. This includes good test–retest reliability (range 0.79 to 0.84), good internal consistency (Cronbach’s alpha between α = 0.66 and 0.92) and validity of therapist ratings [73]. In the present study, overall internal consistency was α = 0.91.

#### Attachment

Utilizing the Revised Adult Attachment Scale (AAS; 29), we assessed attachment insecurity, using the three sub-scales anxiety, closeness, and dependence. This self-report scale consists of 18 items, answered on a 5-point Likert scale (1 – 5; range 18 – 90 with higher scores indicating higher expressions in the respective dimension). The AAS previously demonstrated reliability scores between α = 0.74 and α = 0.86 [74], and very good validity properties [75]. In the present study, internal consistency was α = 0.91.

#### Perceived social support

We measured subjective social support by the Multidimensional Scale of Perceived Social Support (MSPSS; [76]), a 12-item self-report scale. Items are answered on a 7-point Likert scale (1 – 7; range 12 – 84 with higher scores indicating higher perceived support). There are three subscales: perceived support from family, friends, and a significant other. The scale demonstrated very good internal consistency (α = 0.93 to 0.98) and good test–retest reliability (between 0.72 and 0.85) [77]. In this study, internal consistency was α = 0.92.

### Data analysis

The sample of participants who completed questionnaires on BPD features and ACE (PAI-BOR, CTQ, *N* = 1682) was included in the estimation of the first network (N1). For the estimation of the second, third, and fourth network (N2, N3, and N4), we used the subset of 1102 individuals from the original sample, who additionally provided information on attachment style and experienced social support (AAS, MSPSS). The CTQ contained missing variables for the sexual abuse and physical neglect subscales. If there were not more than one missing item per subscale, we did not exclude the data to avoid information loss.

#### Network structure

We estimated two main networks. In the first one (N1), we investigated the interplay of BPD features (four PAI-BOR subscales) and types of ACE (five CTQ subscales). In the second one, we additionally took attachment insecurity (three AAS subscales) and perceived social support (three MSPSS subscales) into account (N2). Controlling for participants’ age and sex, we came up with a total number of 11 nodes for N1 and 17 nodes for N2. To test if findings for the larger sample were comparable to findings for the smaller subsample, we re-estimated N1 with the smaller subsample that completed all measures (including AAS and MSPSS). We performed a network difference test as implemented in the package *NetworkComparisonTest* [78]. No significant differences in edges and global strength were detected (network invariance test: *p* = 0.984; global strength invariance test: *p* = 0.498; further details about this network can be found in supplementary material N3). Furthermore, we additionally estimated N2 without the influence of BPD features (N4, 13 nodes, see supplementary material). All analyses were calculated with RStudio (Version 1.3.959) using a significance level of α = 0.05.

#### Network estimation

Since we added participants’ sex as a binary variable to the networks of normal and non-normal distributed continuous variables, we had mixed variables. Therefore we decided to deviate from our pre-registration and estimated regularized Mixed Graphical Models using the *mgm* function of the package *mgm* [79] as implemented in the *estimateNetwork* function in the bootnet package [80]. The edges estimated between two nodes indicate the unique association between them after controlling for other nodes in the network. The least absolute shrinkage and selection operator (LASSO, [81]) was used by *mgm* to avoid false-positive findings. The LASSO shrinks all edge weights towards zero, therefore small weights become exactly zero, leading to a sparse network structure. Due to our relatively large sample size, we decided to select the parameter λ, which controls the strength of the penalty, using the Extended Bayesian Information Criterion (EBIC; [82]) with the hyper-parameter set to default (γ = 0.25). The edges estimated between the nodes can be interpreted as partial correlations, calculated via nodewise regressions.

#### Node predictability

Note predictability was calculated to assess the connectivity of the nodes. It measures the amount of variance per node, which can be predicted by its neighbors. Node predictability was calculated using the residual R^2^ error value from the estimation of Mixed Gaussian Models (MGM) on each network using the *mgm* package [79]. It is visually depicted as colored pie chart rings around the nodes. A fully colored ring would mean that the node could be completely predicted given its neighbors.

#### Node strength and bridge strength

We focused on strength and bridge strength centrality of the nodes as centrality indices. In the context of psychological networks, other centrality indices such as betweenness and closeness could be misleading [83, 84] which is why we chose to calculate strength centrality. The strength of a node is the sum of the absolute value of the partial correlations (edges) with the other nodes. The bridge nodes act as central nodes in connecting different communities in the network. The bridge strength of a node is the sum of the absolute edge weights of that node with nodes in other communities. To calculate the bridge strength centrality, communities were defined based on the questionnaires in our study. Nodes of PAI-BOR, CTQ, and the control variables (age and sex) were seen as distinct communities in network N1. In network N2, AAS and MSPSS were defined as additional nodes. Then, the bridge strength values were calculated to find out the most central nodes in the network. To take a closer look at the interplay of the interactions of our four communities, we calculated three separate bridge strength values for each node, each representing the sum of the absolute edge weights connecting that node to one of the other three communities. In addition, we summed up the absolute values of edges per community, which connected this community to one of the others.

#### Network accuracy and stability

To measure the accuracy and robustness of the estimated parameters, accuracy and stability tests using bootstrapping procedures were used. The accuracy of the edge weights was calculated using the non-parametric bootstrap method (for constructing 95% CIs around the edge weights) with 1000 bootstrap runs. Accuracy of the centrality parameters was calculated using the *bootnet* package [80] using case-drop bootstrapping.

To assess whether one edge or node is significantly stronger than the other, bootstrapped tests of difference were calculated for both edge weight and centrality. If the 95% bootstrapped CI did not contain zero, the difference was considered significant. The stability of the centrality estimates could be measured by the Correlation Stability Coefficient (CSC) which indicates the percentage of the sample that can be dropped while still maintaining the minimum correlation (for the centrality values) of 0.7 with that of the original sample. A value of CSC lesser than 0.25 indicates that the data sample is unstable, whereas a value above 0.5 is preferable.

We used qgraph and bootnet packages in R for visualizing the networks and centrality graphs. The arrangement of the nodes in the network follows the Fruchterman-Reingold algorithm [85], which places highly connected nodes in the center of the network and lesser connected nodes towards the periphery. We made use of the average layout of all the networks as the common layout. The nodes which belong to different categories are colored differently for better visualization.

### Preregistration

The main hypotheses were preregistered together with the design and planned analyses (https://aspredicted.org/jj8rj.pdf).

## Results

### Sample characteristics of network N1

In Table 1, means and standard deviations for the different measures in the overall sample (N1) are reported together with the network parameters strength centrality and bridge strength. In this full sample of 1682 individuals, *n* = 256 (15.21%) scored above the cut-off (PAI-BOR total > 37) for clinically relevant BPD features. Based on established cut-offs [70, 73], more than half of the participants reported moderate to severe levels of abuse and neglect (emotional neglect: *n* = 1036, 61.59%; emotional; abuse: *n* = 478, 28.42%; physical abuse: *n* = 812, 48.28%; sexual abuse: *n* = 771, 45.84%; physical neglect: *n* = 244, 14.51%). For further details on childhood traumatization see supplemental material Table S1.

**Table 1:** Sample description with parameters of network inference for N1

Measure	Mean	SD	range	Labelling in NA	St-C	Br-St	*R*^*2*^
Sex	27.76% male			Sex	.71	.56	.72
Age	25.98	8.98	18—65	Age	.90	.75	.13
PAI-BOR total	27	9.00	6—56				
﻿ affective instability	8.22	2.78	2 -16	PAI_AI	.76	.00	.45
﻿ identity disturbance	7.16	3.31	1- 15	PAI_ID	1.17	.39	.53
﻿ negative relationships	7.96	2.89	2—16	PAI_NR	.78	.17	.44
﻿ self-harm	3.69	2.20	1—13	PAI_SH	.61	.21	.29
CTQ total	50.8	12.51	30—107				
﻿ emotional abuse	11.28	5.30	5—25	CTQ_EA	1.48	.98	.45
﻿ physical abuse	9.26	3.99	5—25	CTQ_PA	1.12	.11	.41
﻿ sexual abuse	6.94	3.58	4—20	CTQ_SA	.87	.31	.30
﻿ emotional neglect	15.14	3.53	6 -25	CTQ_EN	.78	.19	.26
physical neglect	8.19	1.84	4—18	CTQ_PN	.18	.00	.03

*N* = 1682; *NA* Network Analysis, *St-C* Strength Centrality, *Br-St* Bridge Strength, *R*^2^ Predictability

### Network estimation N1

**Fig. 1:**
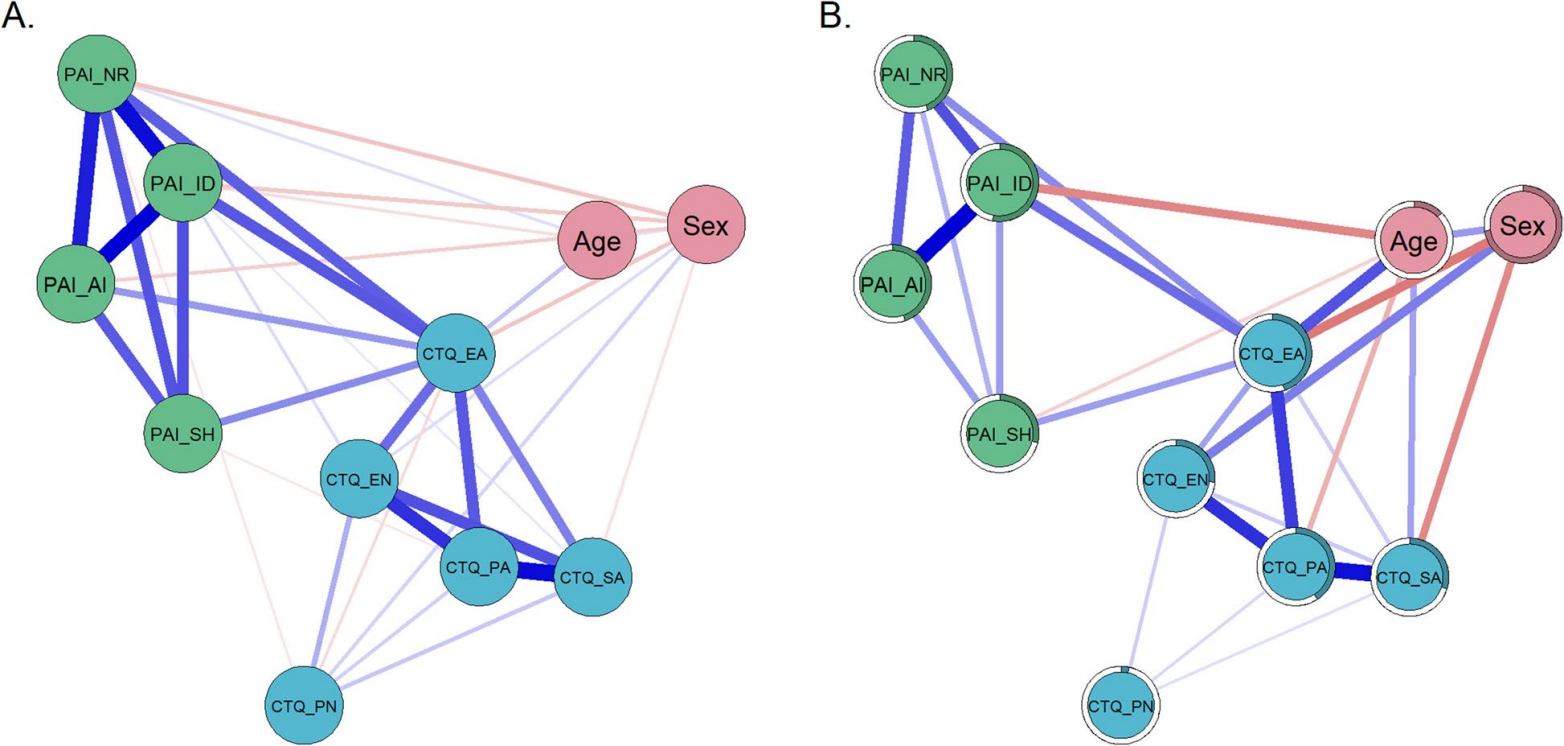
Relations between the elements of network N1. **A** Spearman correlation coefficients. **B** mgm network, estimated via *mgm* with edges signifying unique associations between nodes. Note: In both figures, the thickness of a line indicates the strength of the connection. Blue lines indicate positive associations and red lines indicate negative associations. The colored part of the circular ring around the nodes represents the predictability of the node by its connected nodes (*R*.^2^)

Figure 1A shows Spearman correlation coefficients (*r*_*s*_) between all variables used as nodes in N1 to allow comparisons with studies that report correlation coefficients. Figure 1B displays the estimated mgm network (for more details on Spearman correlation coefficients, edge weights and bootstrapped difference test of edge weights see supplementary material Table S2, Table S3, Figure S3). The mgm network only displays relations between nodes that remained after controlling for all other dependencies in the network. Out of 55 possible edges, 27 (49.09%) had an absolute edge weight above zero (see Fig. 1b, Figure S1b).

#### Centrality estimates N1

Emotional abuse (CTQ_EA score) was the most strongly connected node within N1. It was connected to 8 out of 10 other nodes and its centrality score was significantly higher than that of all other nodes (for centrality values and bootstrapped difference test, please see Figure S1 in the supplemental material). The centrality stability test for node strength revealed a CS-coefficient of 0.75, which is above the recommended value of 0.5. It indicates that if 75% of the cases were dropped, the correlation between the order of resulting centrality strength values and the original order would be at least 0.7 with 95% probability.

#### Bridge strength N1

Emotional abuse was not only the node with the highest centrality strength but also the one with the highest bridge strength (Table 1). This means that it had more and/or stronger inter-cluster edges bridging the theoretically defined clusters of ACE nodes to the group of BPD nodes. In our network, a higher severity of different types of ACE was related to higher severity of BPD features via a higher severity of emotional abuse (for further details on bridge strength and bootstrapped difference test see Figure S2 in the supplementary material). Please note that emotional abuse (CTQ_EA) shows unique associations with the nodes representing identity disturbance, negative relationships, and self-harm, but not with affective instability (PAI_AI). None of the other types of ACE had a unique relation to any the nodes of the BPD domains.

### Sample characteristics of network N2

For the N2 sample, means and standard deviations of all scales together with the network parameters strength centrality and bridge strength are reported in Table 2. In this sub-sample, *n* = 187 (19%) indicated clinically relevant BPD features; emotional neglect was reported by *n* = 605 (54.90%); emotional; abuse: *n* = 390 (35.39%), physical abuse: *n* = 425 (38.57%), sexual abuse: *n* = 399 (36.21%), physical neglect: *n* = 174 (15.79%), see also Table S4 in the supplemental material.

**Table 2:** Sample description with parameters of network inference for N2

Measure	Mean	SD	range	Labelling in NA	St-C	Br-St	*R*^*2*^
Sex	26.32% male			Sex	.3	.28	.74
Age	26.89	9.44	18—65	Age	.36	.28	.14
PAI-BOR total	27.74	8.96	6—56				
﻿ affective instability	8.31	2.67	2 -16	PAI_AI	.63	.00	.42
﻿ identity disturbance	7.34	3.33	1- 15	PAI_ID	1.01	.44	.61
﻿ negative relationships	8.25	2.86	2—16	PAI_NR	.68	.23	.46
﻿ self-harm	3.84	2.28	1—13	PAI_SH	.54	.16	.32
CTQ total	50.25	13.87	30—107				
﻿ emotional abuse	11.91	5.87	5—25	CTQ_EA	1.33	.90	.66
physical abuse	8.73	4.19	5—25	CTQ_PA	.81	.00	.38
﻿ sexual abuse	6.67	3.94	4—20	CTQ_SA	.55	.08	.26
﻿ emotional neglect	14.76	3.71	6 -25	CTQ_EN	.46	.21	.32
﻿ physical neglect	8.17	1.97	4—18	CTQ_PN	.17	.00	.05
AAS							
attachment closeness	19.48	5.82	6 – 30	AAS_C	.52	.08	.45
attachment dependence	16.70	5.46	6 – 30	AAS_D	1.41	.66	.68
attachment anxiety	19.23	6.75	6 – 30	AAS_A	.89	.57	.64
MSPSS total	62.75	16.07	12 – 84				
social support family	19.36	7.50	4 – 28	SS_FA	.96	.71	.54
social support friends	21.22	6.23	4 – 28	SS_FR	.62	.26	.39
social support significant other	28.00	6.53	4 – 28	SS_SO	.62	.20	.28

*n* = 1102; *NA* Network Analysis, *St-C* Strength Centrality, *Br-St* Bridge Strength, *R*^*2*^ Predictability

### N2 network estimation

**Fig. 2:**
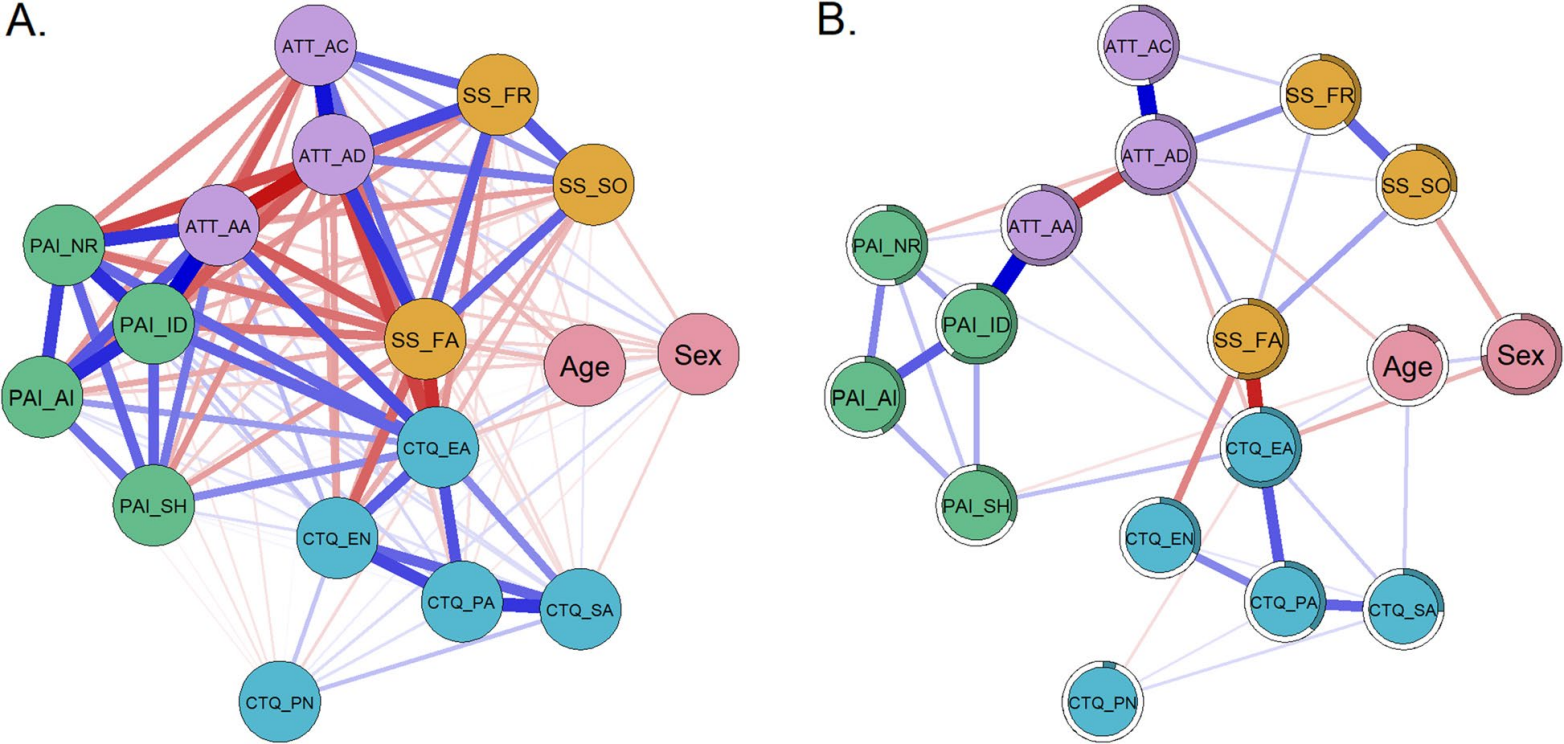
Relations between the elements of network N2. **A** Spearman correlation coefficients. **B** mgm network, estimated via *mgm* with edges signifying unique associations between nodes. Note: In both figures, the thickness of a line indicates the strength of the connection with blue lines indicating positive associations. Red lines indicate negative associations. The colored part of the circular ring around the nodes represents the predictability of the node by its connected nodes (R2)

Figure 2 shows Spearman correlation coefficients between the variables used as nodes in the second network (Fig. 2a) and the estimated mgm network (Fig. 2b). For more details on correlation coefficients and edge weights, see supplementary material Table S5 and S6. In the mgm network analysis, 39 (28.68%) out of 136 possible edges had an absolute edge weight above zero (Fig. 2b).

#### Centrality estimates N2

The most strongly connected nodes within the second network were those for the attachment dimension dependence (AAS_AD) and the ACE subtype emotional abuse (CTQ_EA). These nodes had a significantly higher centrality score than the twelve other nodes in the network. Dependence and emotional abuse were connected to eight out of 16 and 10 out of 16 other nodes respectively. The centrality strength was, however, not significantly stronger than for the level of identity disturbances (PAI_ID, 4 connections), social support from family (SS_FA, 5 connections), and physical abuse (CTQ_PA, 3 connections). For bootstrapped test of difference, please see Supplemental Figure S4. The centrality stability test for node strength revealed a CS-coefficient of 0.75, which is above the recommended value of 0.5. It indicates that if 75% of the cases were dropped, the correlation between the order of resulting centrality strength values and the original order would be at least 0.7 with a probability of 95%.

#### Bridge strength N2

Since in the second network (N2) we investigated the interplay of four groups of nodes (ACE, borderline features, attachment, and social support), more than one bridge node is of interest. Within each of the communities, bridge strength analysis revealed emotional abuse (CTQ), support from family (SS), attachment dependency and anxiety (AAS) as well as identity disturbance (PAI-BOR) as being the strongest potential bridge nodes, with significantly higher bridge strength than other nodes of their community (bootstrapped difference test in supplemental material Figure S5).

**Fig. 3:**
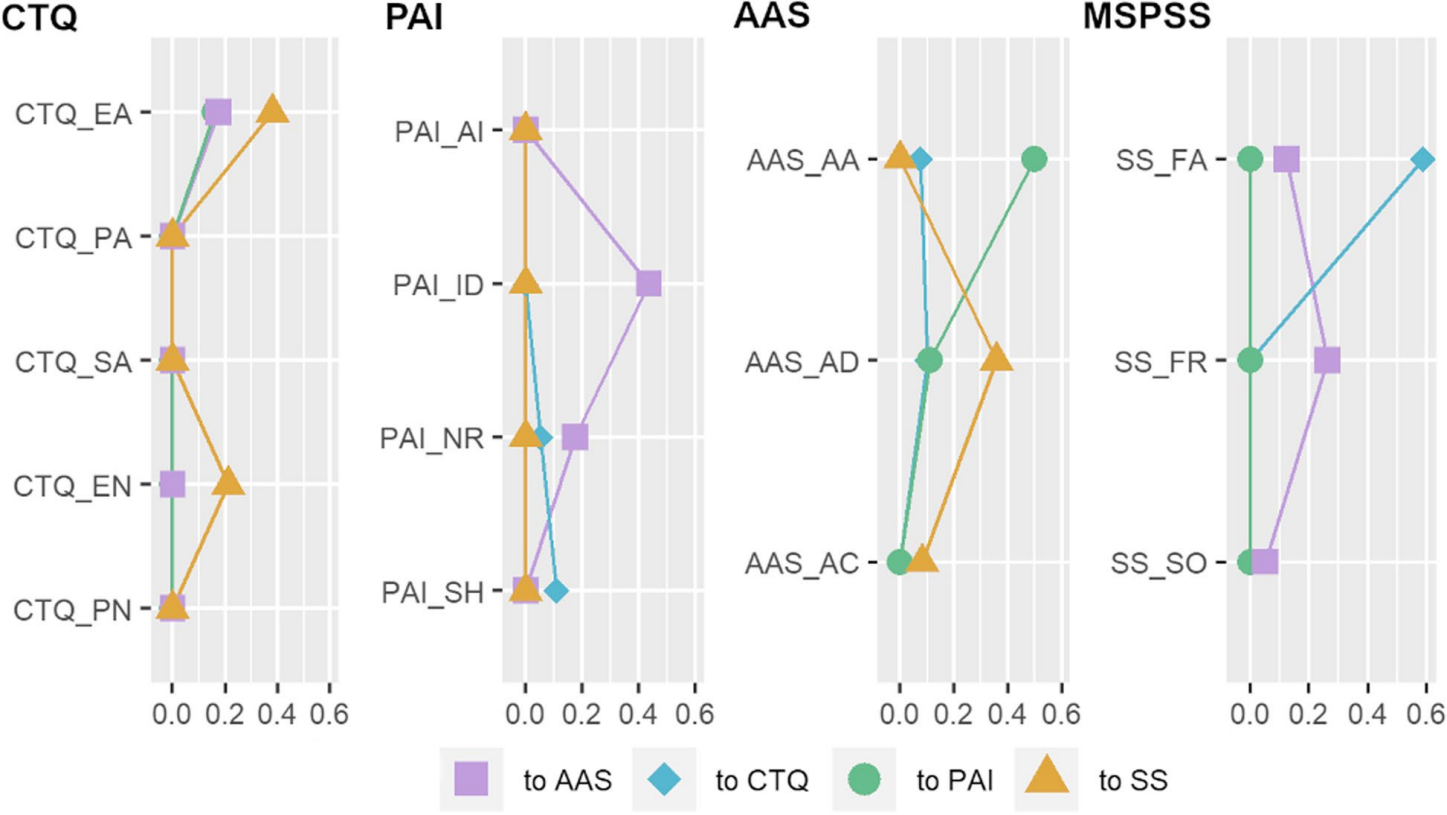
Bridge Strength to different communities in network N2

To investigate the potential pathway from ACE to BPD features on the node level, we first calculated three separate bridge strength values for each node, each representing the sum of the absolute edge weights connecting that node to one of the other three communities (Fig. 3). These analyses revealed that the ACE (CTQ) community was most strongly associated with the perceived social support (MSPSS) community. This association was driven by the strong negative associations between emotional abuse (to a significantly lower extent also emotional neglect) and perceived social support from family members. The social support community was moderately associated with the attachment community, via weak to moderate associations of all three social support nodes to the capacity to feel close to others. The attachment community was strongly associated with the PAI community, via the strong association of attachment anxiety with identity disturbances. Beyond this pathway, the CTQ community was slightly associated with the AAS and PAI communities through emotional abuse. Furthermore, while BPD features were associated with ACE and attachment, there were no unique associations between social support and BPD features.

To have a closer look at the general interplay of our four communities, we summed up the absolute values of edges per community, which connected it to one of the others (Table 3). The CTQ and MSPSS communities were closely connected and so were the AAS and PAI communities. In addition, there was a moderate connection between the MSPSS and AAS communities and slight connections from the CTQ community to the PAI and AAS community respectively. For more details about nodewise bridge strength to different communities see Table S7 in the supplemental material.

**Table 3:** Total absolute inter-community associations N2

	CTQ	PAI	AAS
CTQ			
PAI	.160		
AAS	.174	.609	
MSPSS	.589	0	.358

Sum of absolute edge weights between communities

*MSPSS* Multidimensional Scale of Perceived Social Support

#### Further edge weights of interest in network N2

When taking attachment and perceived social support into account, the positive link from emotional abuse to BPD negative relationships and self-harm remained significant, while the link to identity disturbances (observed in N1) did not. Attachment anxiety was not itself related to social support, but negative links were found through a lower capacity to feel close to others. For further details on bootstrapped edge weights and difference, test see Figure S6 in the supplementary material.

## Discussion

In the present study, we investigated associations between different types of ACE (emotional, physical, and sexual abuse, and neglect) and BPD features (affective instability, identity disturbance, self-harming impulsivity, and relationship problems) as well as the role of attachment and perceived social support in this context, using a graph-theoretical approach. Our main findings were: 1) Emotional abuse was an important hub connecting different types of ACE to different BPD subdomains. 2) The relationship between ACE and BPD features was partially but not fully explained by attachment and social support. 3) In contrast to our hypotheses, perceived social support was not specifically associated with attachment anxiety. Perceived social support was, however, related to a higher ability/willingness to depend on others. Moreover, identity disturbance played a central role in our networks: In the first network, it indirectly linked affective instability to emotional abuse. In the second network, it was particularly strongly associated with attachment anxiety.

### Associations between ACE and BPD features

Emotional abuse had the highest centrality strength indicating particularly high importance within the first network. Moreover, it was identified as a bridge linking ACE to BPD features. Our first network analysis revealed a conditionally independent association between emotional abuse and all BPD features except affective instability. In other words, associations between emotional abuse and most BPD features (identity disturbance, negative relationships, impulsive self-harm) persisted after taking inter-dependencies with other variables into account. The relationship between emotional abuse and different domains of BPD was comparably strong for all BPD features (identity disturbance, negative relationships, and self-harm) except affective instability. There were no direct associations between other ACE (emotional and physical neglect, physical and sexual abuse) with BPD domains. In this respect, our findings only partially confirm the first hypothesis that all types of ACE are related to all BPD features, when taking their shared variance into account. This hypothesis was based on previous theories and studies proposing a close association between ACE and BPD (e.g. [7, 8]). Previous studies found significant associations of BPD with other types of ACE, including emotional neglect [8–10] and sexual abuse [8, 11–15]. While these discrepancies may be partly due to differences in sample characteristics and study designs, our study emphasizes the importance of emotional abuse. Emotional abuse involves experiences of social rejection, as well as emotional invalidation and devaluation by caregivers [86]. Our data suggest that these interactional patterns are of particular importance for understanding the severity of BPD features.

Interestingly, identity disturbance had the highest centrality strength among all four BPD features. At first, this finding may be surprising, as affective instability emerged as a central node in previous network analyses [61, 63, 87]. Affective instability is also a main clinical focus and diagnostic criterion for BPD [88]. In the network analysis by Southward and Cheavens (2018), intense and instable mood as well as chronic emptiness had the highest centrality indices. Similarly, Richetin and colleagues (2017) found affective instability to be a relatively central node. Yet, identity disturbance and efforts to avoid abandonment showed comparably high estimated centrality indices. Moreover, both affective instability and identity disturbances were relatively central nodes in the most recent network analysis by Peters and colleagues [85]. In our study, identity disturbance was more strongly connected to affective instability than other BPD features. This may suggest that identity disturbance might indirectly link affective instability to experiences of emotional abuse. Overall, these findings highlight the importance of identify diffusion and instable self in the context of BPD. This is in line with the current Alternative Model of Personality Disorders (AMPD) in DSM-5, which proposes that deficits in personality functioning are an important component of personality disorders [89, 90]. Maladaptive self and identity function is amongst these proposed deficits [87], which has been supported by recent research [91, 92].

### The role of attachment and social support in associations between ACE and BPD

To investigate the role of attachment and perceived social support in the interplay of BPD and ACE, we extended the network by nodes representing these facets. When taking attachment and perceived social support into account, emotional abuse was still related to the BPD features domains of negative relationships and self-harm. However, there was no longer a direct association between emotional abuse and identity disturbances. It is possible that this is due to a lower number of participants and increased number of nodes in N2, which reduced statistical power. However, a recalculation of the first network with this smaller subsample (participants included in N2) resulted in edges that did not differ from those of the original N1. Therefore, our finding might be interpreted in favor of a significant influence of attachment and social support. This would be in line with prior literature, suggesting that insecure attachment may link ACE to BPD features [23, 24]. Our results also emphasize prior findings, which found loneliness to be an important factor in the development of mental disorders in individuals who experienced ACE [50]. Overall, our analyses confirmed our second hypothesis that attachment dimensions and social support explain part of the variance, but do not fully explain the interrelationship between ACE and BPD.

### Associations of attachment and social support

Attachment and perceived social support were moderately associated with each other. This is in line with earlier findings [25, 31]. We did not find a direct negative link between attachment anxiety and perceived social support. However, our network revealed an indirect negative link between attachment anxiety and perceived social support via the capacity to depend on others. Dismissal of emotional dependence and closeness are key elements of an avoidant attachment style [30], which has been associated with a denial of distress and unwillingness to seek support [41, 42]. In this context, our findings are in line with previous research suggesting that insecurely attached individuals may be less likely to seek and find support or comfort in their social relationships [36, 43].

### The interplay of ACE, social support, attachment, and BPD

A more detailed look at network N2 revealed that there were two pairs of connected networks, that is, ACE and perceived social support as well as BPD features and attachment. The first pair, comprising ACE and perceived social support, contained particularly strong associations between emotional abuse and neglect with perceived family support. Those who reported more emotional maltreatment also evaluated family members as less supportive. At the first glance this finding may not be surprising as these associations may be partly explained by the conceptual overlap of the measures. Items on perceived social support mainly refer to emotional support. If family members were retrospectively experienced as emotionally neglectful or abusive, current support from family may also be perceived as low. Interestingly, however, there was no direct relation between ACE and perceived social support from friends and a significant other. These were only indirectly related through perceived family support. This suggests that ACE might not necessarily influence the perception of current relationships outside the family, despite being slightly connected to perceived family support in the past and now.

The second pair of communities was formed by BPD features and attachment dimensions, driven by a strong association between identity disturbances and attachment anxiety. Fears of being left alone or abandoned may be associated with a greater focus on others, which may, in turn, hinder the development of a stable sense of identity. This may create a vicious cycle, which is in line with the assumption that one's attachment history constantly serves as a basis for identity formation [93]. Earlier conceptualizations have proposed that disturbed interactions with caregivers and insecure attachment are risk factors for identity diffusion as a central element of BPD [51].

Additional weak but unique links were found between both attachment dependency and attachment anxiety and negative relationships. In other words, attachment was indeed associated with difficulties in relationships in our sample, similar to what has been found in other studies [94, 95]. Self-harm and affective instability were only indirectly associated with attachment, through an unstable self-image and difficulties in relationships.

While there were no unique associations between BPD features and any facet of perceived social support, both were indirectly linked via attachment. This is in line with prior literature that revealed strong associations between insecure attachment and severity of BPD features [3, 23, 25] and suggests that insecure attachment influences the subjective perception of social support [36]. To elucidate if insecure attachment may provide a pathway between social support and BPD features, future studies with prospective designs are needed.

In addition, emotional abuse was the only type of ACE that showed unique associations with attachment dimensions. This finding again points to an important role of emotional abuse being the node with the highest bridge strength in the ACE community, both in the first and second network.

### Limitations

To the best of our knowledge, our study is the first that integrated different types of ACE, subdomains of BPD, dimensions of attachment, and perceived social support in one network analytical model. Findings need to be interpreted in the light of several limitations. First, this is a cross-sectional study relying on participants’ subjective reports. Recruitment was done via an international mental health online platform. While this allowed to assemble a relatively large and diverse international sample, it also required the use of self-report instruments with the accompanying limitations. Especially with regards to ACE, a recent study [96] revealed strong discrepancies between prospective and retrospective measures of ACE emphasizing that the CTQ – albeit a well-established measurement – has to be used with caution. Women were overrepresented in the current sample, highlighting the need to replicate our findings in a larger sample of men. We did not verify the presence or absence of clinical BPD diagnosis, which can be seen as a limitation. At the same time, a dimensional assessment of BPD features may offer additional insights into important domains of personality functioning that may not be fully captured by categorical approaches [89, 90]. Therefore, our study contributes to the current development of a research field focusing on a shift from categorical to dimensional models of psychopathology. Nonetheless, a replication of our network in well- characterized clinical samples would complement our findings and clarify whether the observed interrelationships are specific to BPD or constitute a trans-diagnostically relevant pattern. In this regard, future studies should extend the network model by adding variables that might offer a deeper understanding of the underlying mechanism or modulating factors, such as post-traumatic distress.

Both the cross-sectional approach of the study as well as the network analyses reporting partial correlations do not allow causal conclusions. Proposed pathways need to be investigated in future studies with prospective designs, e.g., to determine whether emotional abuse results in affective instability through a negative self-image or unstable sense of identity. From a network analytical view, the regularization via LASSO, which we used to avoid false positives, may have caused the network structure found to be sparser than the underlying true model might be [97]. Thus, associations that are not mapped in our networks should not automatically be considered as irrelevant.

## Conclusion

Overall, our findings suggest an important role of emotional abuse being a potential bridge in the relationship between ACE and BPD features. This association seems to be partly but not completely explained by social support and attachment. While previous networks mostly revealed affective instability as a central feature in BPD, our results point to an important role of identity disturbances, particularly in connection with insecure attachment. Future studies are needed to deepen the understanding of this interplay and to derive implications for the treatment of interpersonal impairments in BPD.

## Supplementary Information

**Additional file 1: Table S1.** Frequencies of severity categories of childhood maltreatment of N1 sample. **Table S2.** Spearman Correlations of Nodes in Network N1. **Table S3.** Edge Weights in Network N1. **Fig. S1.** Centrality values and bootstrapped difference test of network N1. **Fig. S2.** Bridge strength and bootstrapped difference test of network N1. **Fig. S3.** Bootstrapped Difference Test of Edge Weights N1. **Table S4.** Frequencies of severity categories of childhood maltreatment of N2 sample. **Table S5.** Spearman Correlations of Nodes in Network N2. **Table S6.** Edge Weights in Network N2. **Table S7.** Bridge Strength to Different Communities in Network N2. **Fig. S4.** Centrality values and bootstrapped difference test of network N2. **Fig. S5.** Bridge strength and bootstrapped difference test of network N2. **Fig. S6.** Bootstrapped Difference Test of Edge Weights N2. **Fig. S7.** Relations between the elements of network N3. A. Spearman correlation coefficients. B. Regularized partial correlation network, estimated via mgm with edges signifing unique associations between nodes. Note: In both figures the thickness of a line indicates the strength of the connection with blue colour indicating positive correlation and red the negative ones. The coloured part of the circular ring around the nodes represents the predictability of the node by its connected nodes (R2). **Table S8.** Spearman Correlations of Nodes in Network N3. **Table S9.** Edge Weights in Network N3. **Fig. S8.** Centrality values and bootstrapped difference test of network N3. **Fig. S9.** Bridge strength and bootstrapped difference test of network N3. **Fig. S10.** Bootstrapped Difference Test of Edge Weights N3. **Table S10.** Parameters of Network Inference for N4. **Fig. S11.** Relations between the elements of network N4. A. Spearman correlation coefficients. B. Regularized partial correlation network, estimated via mgm with edges signifing unique associations between nodes. Note: In both figures the thickness of a line indicates the strength of the connection with blue colour indicating positive correlation and red the negative ones. The coloured part of the circular ring around the nodes represents the predictability of the node by its connected nodes (R2). **Table S11.** Spearman Correlations of Nodes in Network N4. **Table S12.** Edge Weights in Network N4. **Fig. S12.** Centrality values and bootstrapped difference test of network N4. **Fig. S13.** Bridge Strength and bootstrapped difference test of network N4. **Fig. S14.** Edge Weights and bootstrapped difference test of network N4.

## Data Availability

According to European law (GDPR), data containing potentially identifying or sensitive patient information are restricted; our data involving clinical participants are not freely available in the manuscript, supplemental files, or in a public repository. Data access can be requested on reasonable demand via A.KU.

## Abbreviations

ACE: Adverse childhood experiences
BPD: Borderline personality disorder
CTQ: Childhood Trauma Questionnaire
CTQ_EA: Emotional abuse
CTQ_PA: Physical abuse
CTQ_SA: Sexual abuse
CTQ_EN: Emotional neglect
CTQ_PN: Physical neglect
PAI-BOR: Borderline Scale from the Personality Assessment Inventory
PAI_AI: Affective instability
PAI_ID: Identity disturbance
PAI_NR: Negative relationships
PAI_SH: Self-harm
AAS: Adult Attachment Scale
AAS_A: Attachment anxiety
AAS_C: Attachment closeness
AAS_D: Attachment dependence
MSPSS: Multidimensional Scale of Perceived Social Support
SS_FA: Support from family members
SS_FR: Support from friends
SS_SO: Support from a significant other
CSC: Correlation Stability Coefficient
