# Supplementary material for: On the interplay of borderline personality features, childhood trauma severity, attachment types, and social support
Source: Borderline Personal Disord Emot Dysregul. 2022 Dec 19;9:35. doi: 10.1186/s40479-022-00206-9 (PMC9762015; doi:10.1186/s40479-022-00206-9)
Supplement: Supplementary file 1 — Additional file 1: Table S1. Frequencies of severity categories of childhood maltreatment of N1 sample. Table S2. Spearman Correlations of Nodes in Network N1. Table S3. Edge Weights in Network N1. Fig. S1. Centrality values and bootstrapped difference test of network N1. Fig. S2. Bridge strength and bootstrapped difference test of network N1. Fig. S3. Bootstrapped Difference Test of Edge Weights N1. Table S4. Frequencies of severity categories of childhood maltreatment of N2 sample. Table S5. Spearman Correlations of Nodes in Network N2. Table S6. Edge Weights in Network N2. Table S7. Bridge Strength to Different Communities in Network N2. Fig. S4. Centrality values and bootstrapped difference test of network N2. Fig. S5. Bridge strength and bootstrapped difference test of network N2. Fig. S6. Bootstrapped Difference Test of Edge Weights N2. Fig. S7. Relations between the elements of network N3. A. Spearman correlation coefficients. B. Regularized partial correlation network, estimated via mgm with edges signifing unique associations between nodes. Note: In both figures the thickness of a line indicates the strength of the connection with blue colour indicating positive correlation and red the negative ones. The coloured part of the circular ring around the nodes represents the predictability of the node by its connected nodes (R2). Table S8. Spearman Correlations of Nodes in Network N3. Table S9. Edge Weights in Network N3. Fig. S8. Centrality values and bootstrapped difference test of network N3. Fig. S9. Bridge strength and bootstrapped difference test of network N3. Fig. S10. Bootstrapped Difference Test of Edge Weights N3. Table S10. Parameters of Network Inference for N4. Fig. S11. Relations between the elements of network N4. A. Spearman correlation coefficients. B. Regularized partial correlation network, estimated via mgm with edges signifing unique associations between nodes. Note: In both figures the thickness of a line indicates the strength of the conn [file 40479_2022_206_MOESM1_ESM.pdf]

Supplementary Material

On the Interplay between Borderline Personality Features, Childhood Trauma, Attachment Types, and Social Support

Anna Schulze [1], Leonie Cloos [2,3], Monika Zdravkovic[2], Stefanie Lis [1,4] *, Annegret Krause-Utz [2] *

* contributed equally

[1] Department of Clinical Psychology, Central Institute of Mental Health, Mannheim, Germany; Medical Faculty, University of Heidelberg, Mannheim, Germany

[2] Institute of Clinical Psychology, Leiden University, Leiden, The Netherlands

[3] Research Group of Quantitative Psychology and Individual Differences, Faculty of Psychology and Educational Sciences, KU Leuven, Belgium

[4] Department of Psychosomatic Medicine and Psychotherapy, Central Institute of Mental Health, Mannheim, Germany; Medical Faculty, University of Heidelberg, Mannheim, Germany

Supplementary Material Network N1

Table S1

Frequencies of severity categories of childhood maltreatment of N1 sample

	none to minimal N (%)	slight to moderate N (%)	moderate to severe N (%)	severe to extreme N (%)
emotional abuse	525 (31.21)	679 (40.37)	142 (8.44)	336 (19.98)
physical abuse	716 (42.57)	154 (9.16)	442 (26.28)	370 (22.00)
sexual abuse	761 (45.24)	150 (8.92)	639 (37.99)	132 (7.85)
emotional neglect	164 (9.75)	482 (28.66)	599 (35.61)	437 (25.98)
physical neglect	366 (21.76)	1072 (63.73)	190 (11.30)	54 (3.21)

Note. N = 1682

Table S2

Spearman Correlations of Nodes in Network N1

	Sex	Age	PAI_AI	PAI_ID	PAI_NR	PAI_SH	CTQ_EA	CTQ_PA	CTQ_SA	CTQ_EN	CTQ_PN
Sex		.006	.000	.000	.000	.413	.000	.339	.005	.001	.000
Age	.068		.491	.001	.001	.725	.000	.130	.285	.103	.162
PAI_AI	-.116	-.017		.000	.000	.000	.000	.122	.110	.468	.280
PAI_ID	-.126	-.079	.615		.000	.000	.000	.331	.014	.000	.070
PAI_NR	-.137	.078	.544	.583		.000	.000	.899	.510	.071	.014
PAI_SH	-.020	.009	.397	.420	.413		.000	.021	.285	.251	.175
CTQ_EA	-.134	.143	.249	.403	.388	.279		.000	.000	.000	.000
CTQ_PA	-.023	-.037	-.038	.024	.003	-.056	.401		.000	.000	.000
CTQ_SA	-.068	.026	.039	.060	.016	-.026	.305	.592		.000	.000
CTQ_EN	.082	.040	.018	.089	.044	.028	.355	.497	.419		.000
CTQ_PN	.103	.034	-.026	-.044	-.060	-.033	-.085	.111	.132	.192	

Note. Spearman’s correlation coefficients (below diagonal) and *p*-values (above diagonal)

Table S3

Edge Weights in Network N1

	Sex	Age	PAI_AI	PAI_ID	PAI_NR	PAI_SH	CTQ_EA	CTQ_PA	CTQ_SA	CTQ_EN
Age	.149									
PAI_AI	.000	.000								
PAI_ID	.000	-.177	.376							
PAI_NR	.000	.000	.237	.257						
PAI_SH	.000	-.065	.144	.143	.114					
CTQ_EA	-.196	.254	.000	.215	.172	.143				
CTQ_PA	.000	-.114	.000	.000	.000	.000	.286			
CTQ_SA	-.174	.138	.000	.000	.000	.000	.074	.353		
CTQ_EN	.191	.000	.000	.000	.000	.000	.138	.306	.079	
CTQ_PN	.000	.000	.000	.000	.000	.000	.000	.056	.046	.073

Note. Partial correlation coefficients.

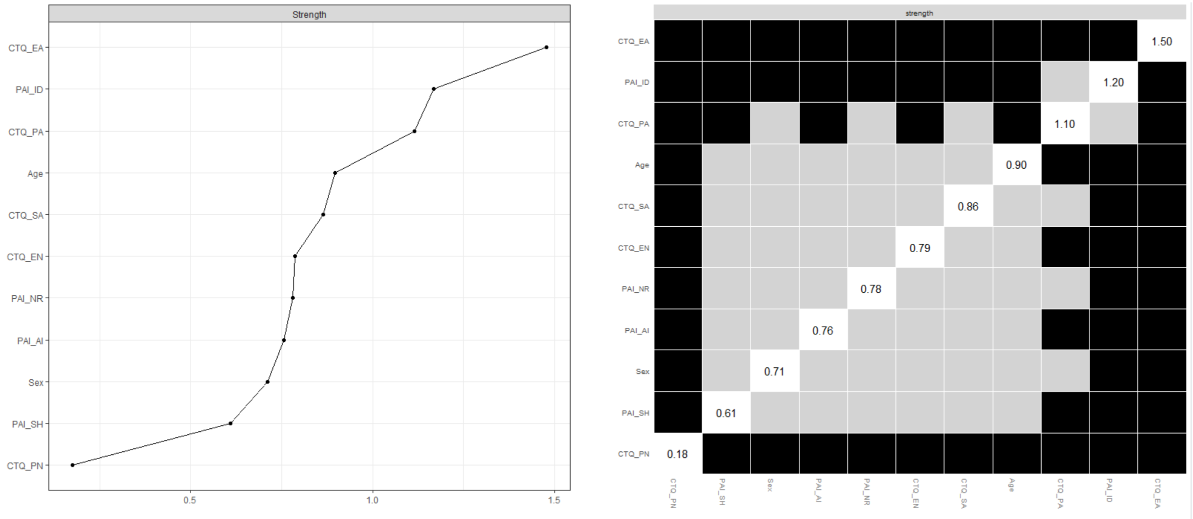

Fig. S1.Centrality values and bootstrapped difference test of network N1

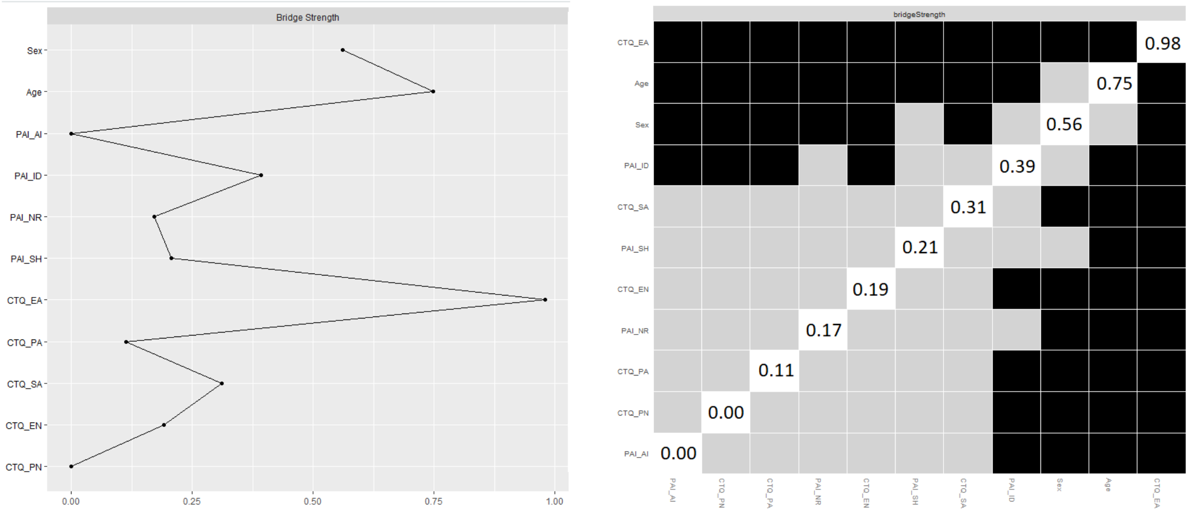

Fig. S2. Bridge strength and bootstrapped difference test of network N1

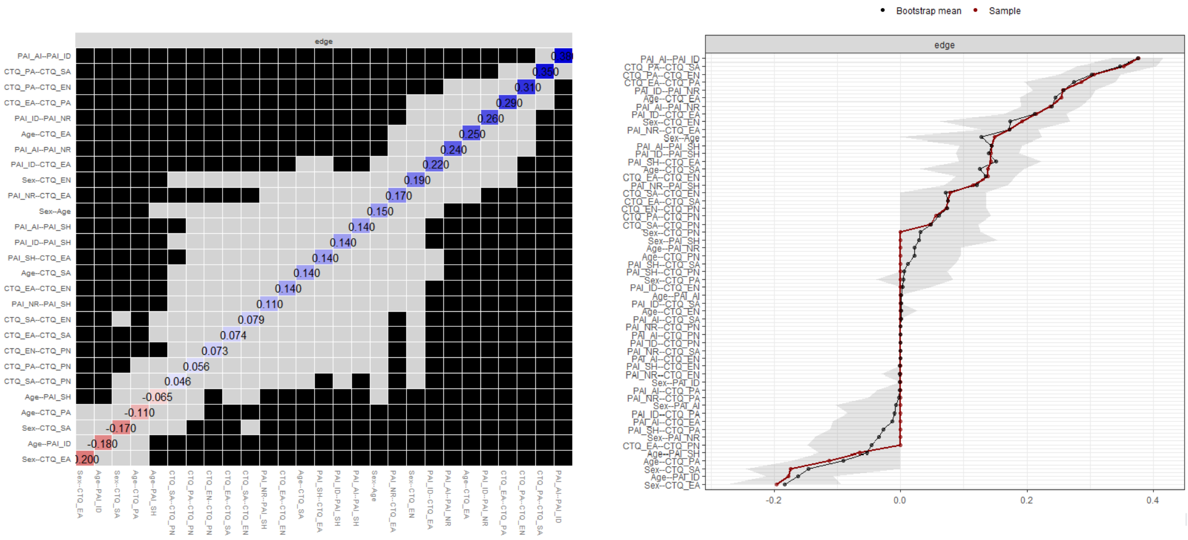

Fig. S3. Bootstrapped Difference Test of Edge Weights N1

Supplementary Material Network N2

Table S4

Frequencies of severity categories of childhood maltreatment of N2 sample

	none to minimal N (%)	slight to moderate N (%)	moderate to severe N (%)	severe to extreme N (%)
emotional abuse	366 (33.21)	346 (31.40)	105 (9.53)	285 (25.86)
physical abuse	569 (51.63)	108 (9.80)	224 (20.33)	201 (18.24)
sexual abuse	613 (55.63)	88 (7.80)	301 (27.31)	98 (8.89)
emotional neglect	131 (11.89)	366 (33.21)	329 (29.85)	276 (25.04)
physical neglect	281 (25.50)	647 (58.71)	132 (11.98)	42 (3.81)

Note. N = 1102.

Table S5

Spearman Correlations of Nodes in Network N2

	Sex	Age	PAI_ AI	PAI_ ID	PAI_ NR	PAI_ SH	CTQ_ EA	CTQ_ PA	CTQ_ SA	CTQ_ EN	CTQ_ PN	ATT_ AC	ATT_ AD	ATT_ AA	SS_ FA	SS_ FR	SS_ FR
Sex		.035	.000	.000	.000	.308	.000	.053	.000	.000	.002	.005	.000	.000	.661	.926	.000
Age	.063		.837	.002	.036	.636	.000	.790	.019	.028	.091	.000	.000	.921	.000	.000	.070
PAI AI	-.133	.007		.000	.000	.000	.000	.418	.006	.018	.166	.000	.000	.000	.000	.000	.000
PAI ID	-.125	-.091	.598		.000	.000	.000	.001	.002	.000	.019	.000	.000	.000	.000	.000	.000
PAI _NR	-.136	.064	.536	.587		.000	.000	.000	.002	.000	.015	.000	.000	.000	.000	.000	.000
PAI SH	-.031	-.014	.396	.439	.431		.000	.414	.319	.002	.258	.000	.000	.000	.000	.000	.000
CTQ EA	-.153	.153	.280	.433	.430	.330		.000	.000	.000	.000	.000	.000	.000	.000	.000	.000
CTQ _PA	-.060	-.008	.025	.104	.129	.025	.486		.000	.000	.000	.000	.000	.000	.000	.003	.014
CTQ SA	-.114	.071	.084	.094	.097	.030	.327	.565		.000	.000	.001	.005	.140	.003	.011	.935
CTQ EN	.038	.065	.070	.168	.127	.094	.455	.514	.434		.000	.000	.000	.000	.000	.000	.000
CTQ _PN	.095	.051	-.042	-.071	-.073	-.034	-.106	.090	.176	.162		.962	.664	.013	.926	.869	.363
ATT AC	.085	-.130	-.283	-.337	-.328	-.200	-.395	-.126	-.101	-.248	-.001		.000	.000	.000	.000	.000
ATT AD	.116	-.200	-.360	-.463	-.512	-.307	-.530	-.150	-.086	-.265	.013	.666		.000	.000	.000	.000
ATT AA	-.135	.004	.477	.713	.570	.383	.500	.112	.046	.193	-.075	-.456	-.655		.000	.000	.000
SS FA	-.016	-.189	-.234	-.378	-.391	-.272	-.581	-.175	-.089	-.432	.003	.423	.558	-.453		.000	.000
SS FR	.000	-.110	-.183	-.298	-.293	-.142	-.294	-.087	-.075	-.235	-.005	.432	.520	-.363	.469		.000
SS SO	-.137	-.054	-.119	-.216	-.212	-.107	-.194	-.072	-.001	-.223	-.027	.299	.370	-.267	.441	.464	

Note. Spearman's correlation coefficients (below diagonal) and *p*-values (above diagonal)

Table S6

Edge Weights in Network N2

	Sex	Age	PAI _AI	PAI _ID	PAI _NR	PAI _SH	CTQ _EA	CTQ _PA	CTQ _SA	CTQ _EN	CTQ _PN	ATT _AC	ATT _AD	ATT _AA	SS_FA	SS_FR
Age	.084															
PAI_AI	.000	.000														
PAI_ID	.000	.000	.281													
PAI_NR	.000	.000	.210	.148												
PAI_SH	.000	-.050	.136	.144	.100											
CTQ_EA	-.129	.060	.000	.000	.052	.108										
CTQ_PA	.000	.000	.000	.000	.000	.000	.285									
CTQ_SA	.000	.081	.000	.000	.000	.000	.087	.268								
CTQ_EN	.000	.000	.000	.000	.000	.000	.000	.207	.047							
CTQ_PN	.000	.000	.000	.000	.000	.000	-.056	.048	.063	.000						
ATT_AC	.000	.000	.000	.000	.000	.000	.000	.000	.000	.000	.000					
ATT_AD	.000	-.090	.000	.000	-.109	.000	-.100	.000	.000	.000	.000	.436				
ATT_AA	.000	.000	.000	.436	.064	.000	.074	.000	.000	.000	.000	.000	-.316			
SS_FA	.000	.000	.000	.000	.000	.000	-.379	.000	.000	-.210	.000	.000	.123	.000		
SS_FR	.000	.000	.000	.000	.000	.000	.000	.000	.000	.000	.000	.080	.183	.000	.095	
SS_SO	-.148	.000	.000	.000	.000	.000	.000	.000	.000	.000	.000	.000	.052	.000	.156	.260

Note. Partial correlation coefficients.

Table S7

Bridge Strength to Different Communities in Network N2

	bridge to			
	CTQ	PAI	AAS	MSPSS
CTQ				
EA		.160	.175	.379
PA		.000	.000	.000
SA		.000	.000	.000
EN		.000	.000	.210
PN		.000	.000	.000
PAI				
AI	.000		.000	.000
ID	.000		.436	.000
NR	.052		.174	.000
SH	.108		.000	.000
AAS				
AA	.000	.000		.080
AD	.100	.109		.358
AC	.074	.500		.000
MSPSS				
FA	.589	.000	.123	
FR	.000	.000	.262	
SO	.000	.000	.052	

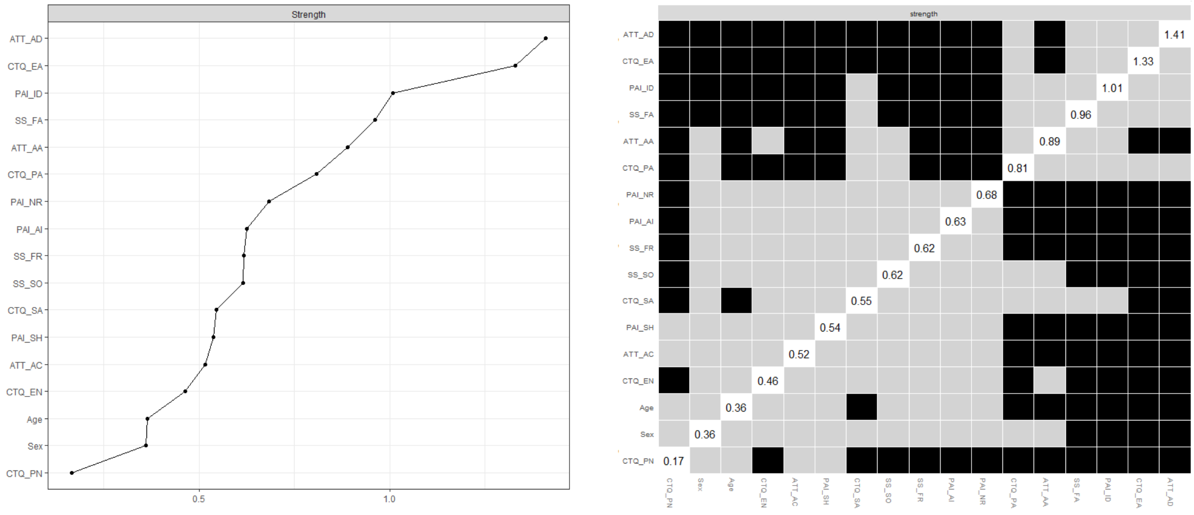

Fig. S4. Centrality values and bootstrapped difference test of network N2

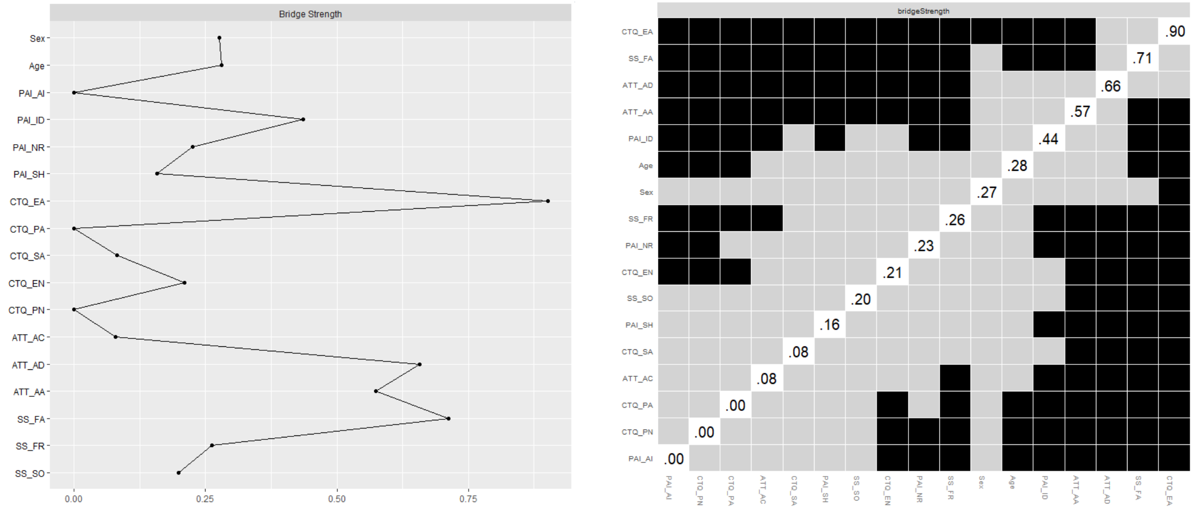

Fig. S5. Bridge strength and bootstrapped difference test of network N2

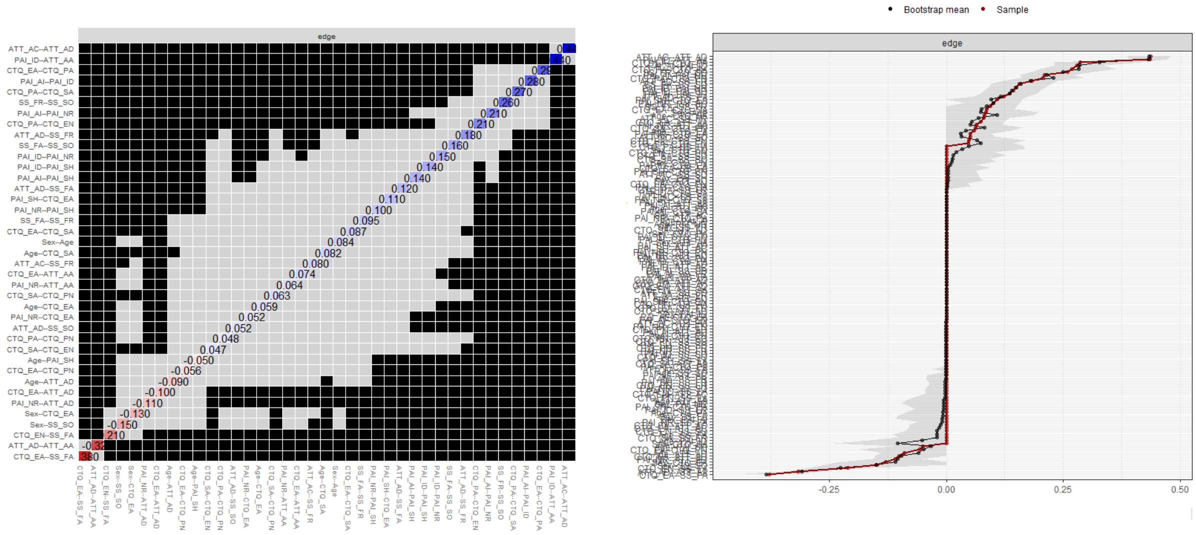

Fig. S6. Bootstrapped Difference Test of Edge Weights N2

Supplementary Material Network N3 (N1 with sample of N2)

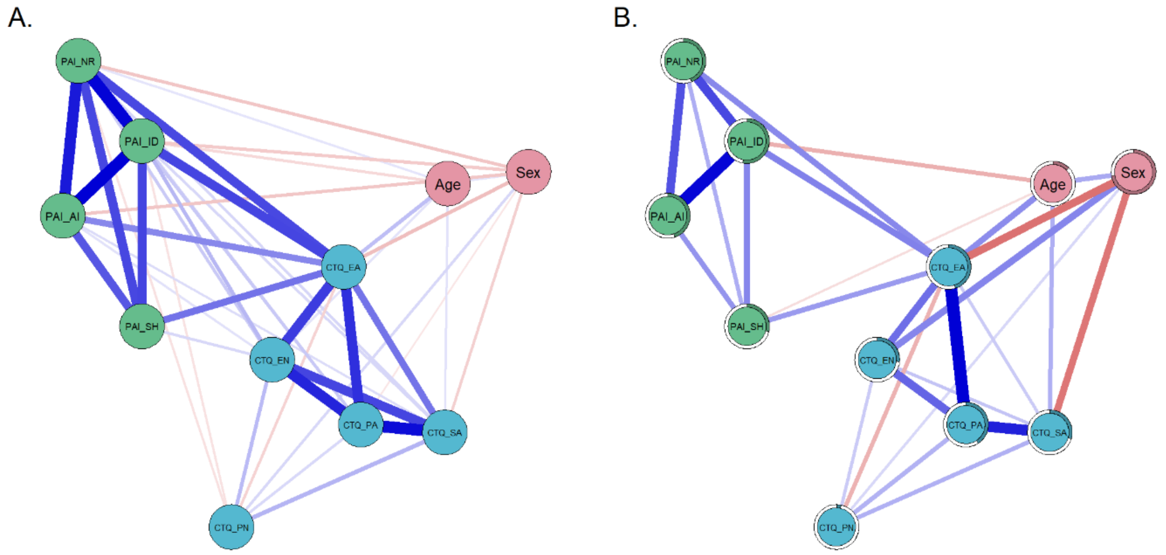

Fig. S7: Relations between the elements of network N3. A. Spearman correlation coefficients. B. Regularized partial correlation network, estimated via *mgm* with edges signifing unique associations between nodes. Note: In both figures the thickness of a line indicates the strength of the connection with blue colour indicating positive correlation and red the negative ones. The coloured part of the circular ring around the nodes represents the predictability of the node by its connected nodes (R^2).

Table S8

Spearman Correlations of Nodes in Network N3

	Sex	Age	PAI_AI	PAI_ID	PAI_NR	PAI_SH	CTQ_EA	CTQ_PA	CTQ_SA	CTQ_EN	CTQ_PN
Sex		.037	.000	.000	.000	.304	.000	.047	.000	.208	.002
Age	.063		.826	.003	.035	.640	.000	.800	.019	.030	.091
PAI_AI	-.133	.000		.000	.000	.000	.000	.400	.006	.021	.165
PAI_ID	-.125	-.071	.600		.000	.000	.000	.001	.002	.000	.019
PAI_NR	-.136	.064	.536	.587		.000	.000	.000	.001	.000	.015
PAI_SH	.000	.000	.396	.439	.431		.000	.409	.316	.002	.258
CTQ_EA	-.153	.153	.280	.433	.430	.330		.000	.000	.000	.000
CTQ_PA	-.060	.000	.000	.104	.129	.000	.486		.000	.000	.003
CTQ_SA	-.114	.071	.084	.094	.097	.000	.328	.565		.000	.000
CTQ_EN	.000	.065	.070	.168	.127	.094	.455	.514	.437		.000
CTQ_PN	.095	.000	.000	-.071	-.073	.000	-.106	.090	.176	.162	

Note. Spearman's correlation coefficients (below diagonal) and *p*-values (above diagonal)

Table S9

Edge Weights in Network N3

	Sex	Age	PAI_AI	PAI_ID	PAI_NR	PAI_SH	CTQ_EA	CTQ_PA	CTQ_SA	CTQ_EN
Age	.118									
PAI_AI	.000	.000								
PAI_ID	.000	-.108	.340							
PAI_NR	.000	.000	.239	.252						
PAI_SH	.000	-.050	.147	.167	.112					
CTQ_EA	-.195	.159	.000	.174	.163	.138				
CTQ_PA	.000	.000	.000	.000	.000	.000	.350			
CTQ_SA	-.189	.110	.000	.000	.000	.000	.073	.305		
CTQ_EN	.167	.000	.000	.000	.000	.000	.199	.213	.082	
CTQ_PN	.055	.000	.000	.000	.000	.000	-.106	.106	.108	.073

Note. Partial correlation coefficients.

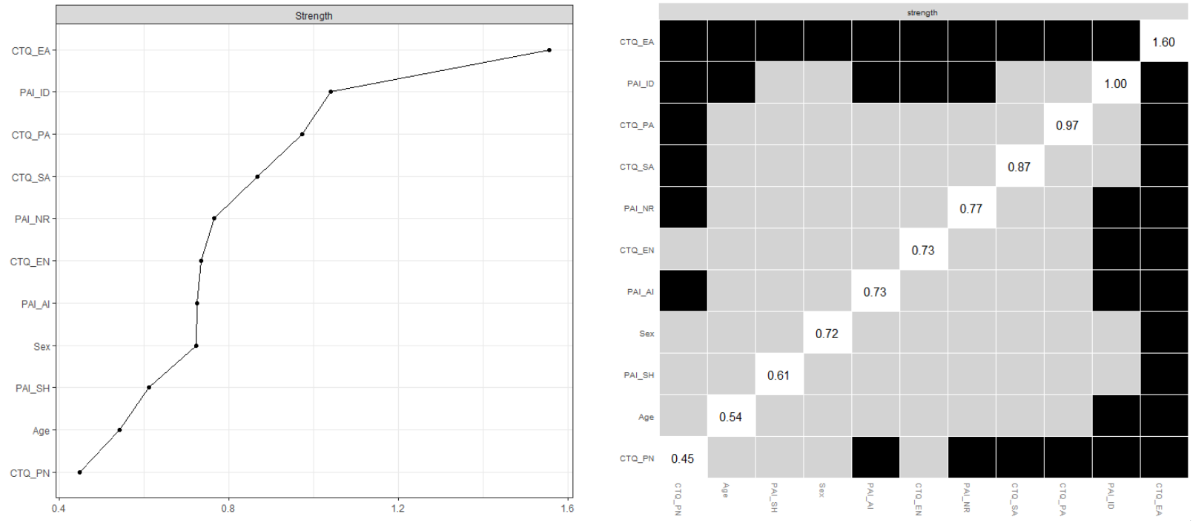

Fig. S8. Centrality values and bootstrapped difference test of network N3

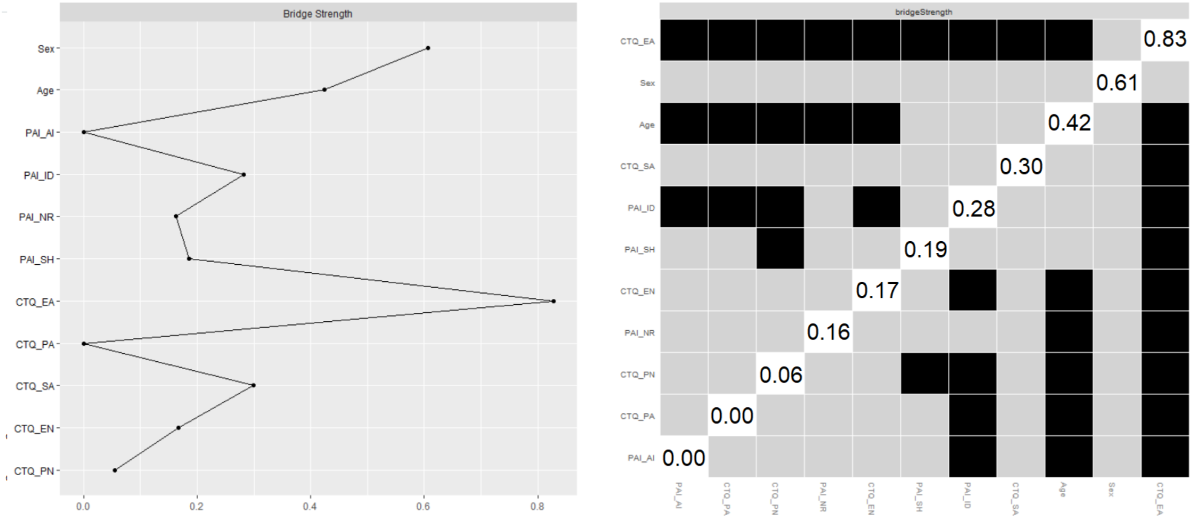

Fig. S9. Bridge strength and bootstrapped difference test of network N3

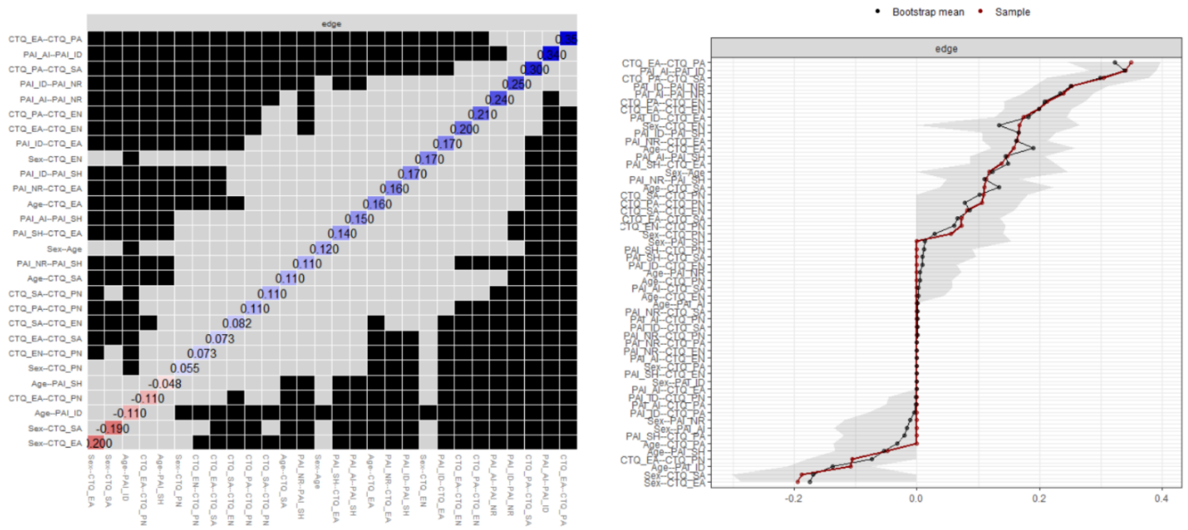

Fig. S10. Bootstrapped Difference Test of Edge Weights N3

Supplementary Material Network N4 (N2 without PAI)

Table S10

Parameters of Network Inference for N4

Measure	Labelling in NA	St-C	Br-St	R^2
Sex	Sex	.531	.445	.743
Age	Age	.453	.368	.102
emotional abuse	CTQ_EA	1.354	.860	.647
physical abuse	CTQ_PA	.906	.000	.411
sexual abuse	CTQ_SA	.851	.304	.309
emotional neglect	CTQ_EN	.504	.210	.315
physical neglect	CTQ_PN	.222	.000	.054
attachment closeness	ATT_AC	.516	.079	.453
attachment dependence	ATT_AD	1.423	.572	.668
attachment anxiety	ATT_AA	.714	.299	.477
social support family	SS_FA	.981	.727	.540
social support friends	SS_FR	.673	.315	.384
social support significant other	SS_SO	.616	.201	.277

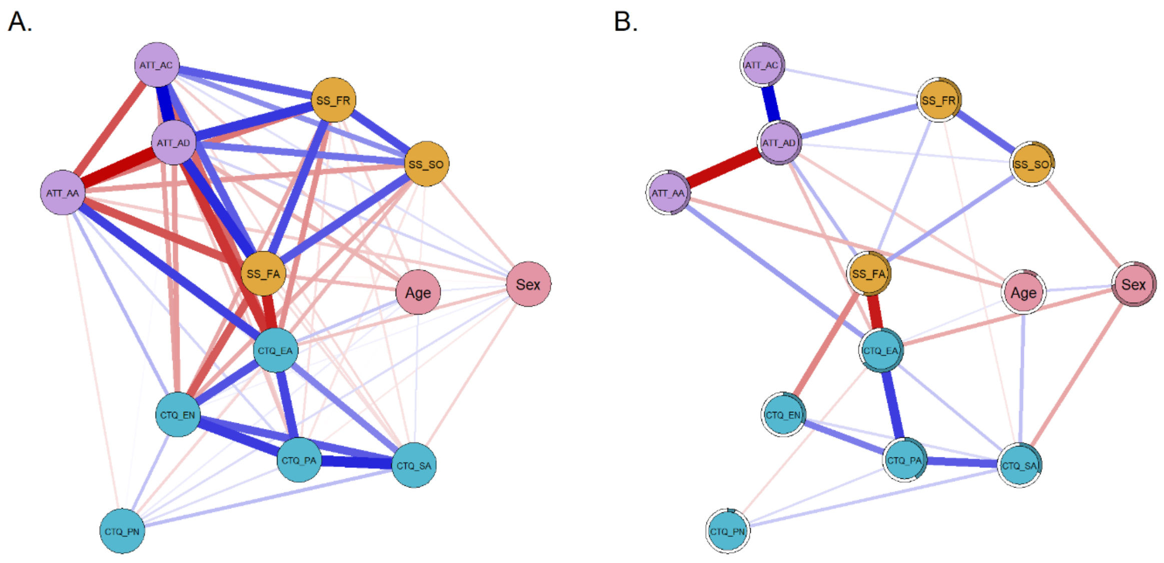

Fig. S11. Relations between the elements of network N4. A. Spearman correlation coefficients. B. Regularized partial correlation network, estimated via *mgm* with edges signifing unique associations between nodes. Note: In both figures the thickness of a line indicates the strength of the connection with blue colour indicating positive correlation and red the negative ones. The coloured part of the circular ring around the nodes represents the predictability of the node by its connected nodes (R^2).

Table S11

Spearman Correlations of Nodes in Network N4

	Sex	Age	CTQ_EA	CTQ_PA	CTQ_SA	CTQ_EN	CTQ_PN	ATT_AC	ATT_AD	ATT_AA	SS_FA	SS_FR	SS_FR
Sex		.035	.000	.053	.000	.000	.002	.005	.000	.000	.661	.926	.000
Age	.063		.000	.790	.019	.028	.091	.000	.000	.921	.000	.000	.070
CTQ_EA	-.153	.153		.000	.000	.000	.000	.000	.000	.000	.000	.000	.000
CTQ_PA	-.060	-.008	.486		.000	.000	.000	.000	.000	.000	.000	.003	.014
CTQ_SA	-.114	.071	.327	.565		.000	.000	.001	.005	.140	.003	.011	.935
CTQ_EN	.038	.065	.455	.514	.434		.000	.000	.000	.000	.000	.000	.000
CTQ_PN	.095	.051	-.106	.090	.176	.162		.962	.664	.013	.926	.869	.363
ATT_AC	.085	-.130	-.395	-.126	-.101	-.248	-.001		.000	.000	.000	.000	.000
ATT_AD	.116	-.200	-.530	-.150	-.086	-.265	.013	.666		.000	.000	.000	.000
ATT_AA	-.135	.004	.500	.112	.046	.193	-.075	-.456	-.655		.000	.000	.000
SS_FA	-.016	-.189	-.581	-.175	-.089	-.432	.003	.423	.558	-.453		.000	.000
SS_FR	.000	-.110	-.294	-.087	-.075	-.235	-.005	.432	.520	-.363	.469		.000
SS_SO	-.137	-.054	-.194	-.072	-.001	-.223	-.027	.299	.370	-.267	.441	.464	

Note. Spearman's correlation coefficients (below diagonal) and *p*-values (above diagonal).

Table S12

Edge Weights in Network N4

	Sex	Age	CTQ_EA	CTQ_PA	CTQ_SA	CTQ_EN	CTQ_PN	ATT_AC	ATT_AD	ATT_AA	SS_FA	SS_FR	SS_FR
Sex													
Age	.085												
CTQ_EA	-.145	.040											
CTQ_PA	.000	.000	.332										
CTQ_SA	-.152	.108	.099	.284									
CTQ_EN	.000	.000	.000	.225	.069								
CTQ_PN	.000	.000	-.063	.064	.095	.000							
ATT_AC	.000	.000	.000	.000	.000	.000	.000						
ATT_AD	.000	-.089	-.113	.000	.000	.000	.000	.437					
ATT_AA	.000	-.130	.169	.000	.000	.000	.000	.000	-.415				
SS_FA	.000	.000	-.392	.000	.000	-.210	.000	.000	.125	.000			
SS_FR	.000	.000	.000	.000	-.044	.000	.000	.079	.192	.000	.098		
SS_SO	-.148	.000	.000	.000	.000	.000	.000	.000	.052	.000	.156	.260	

Note. Partial correlation coefficients.

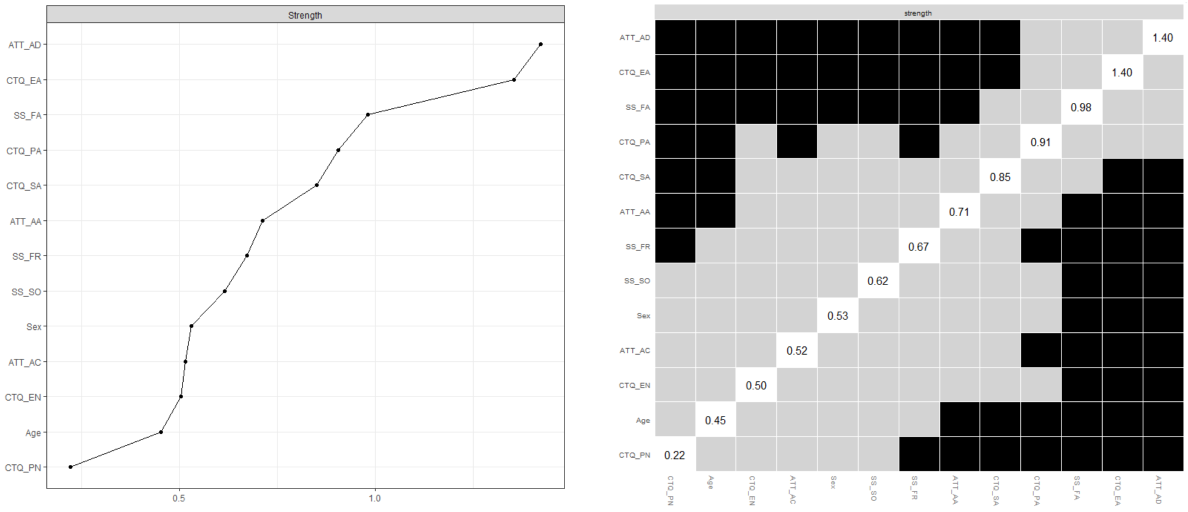

Fig. S12. Centrality values and bootstrapped difference test of network N4.

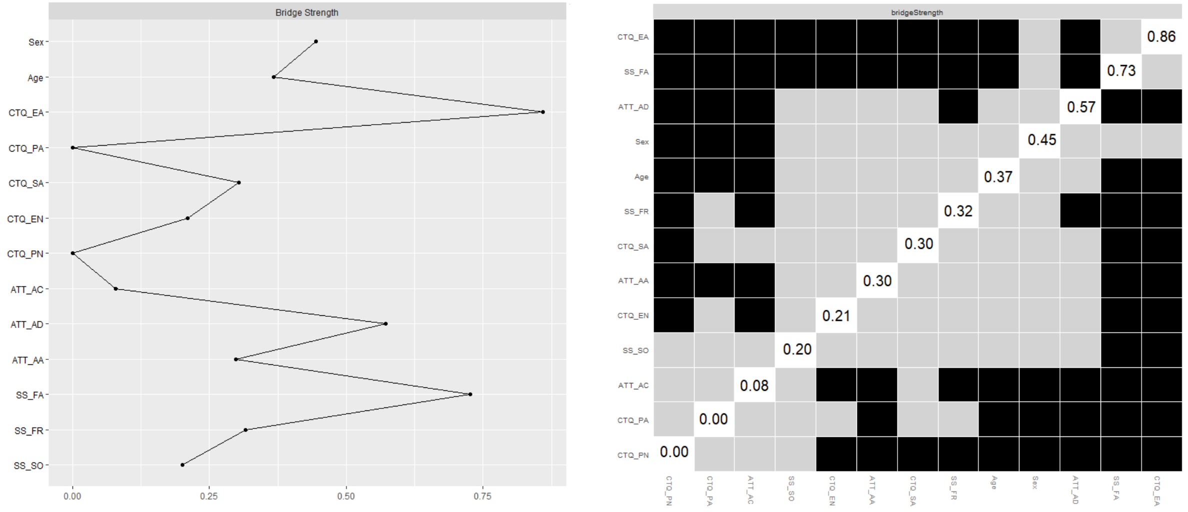

Fig. S13. Bridge Strength and bootstrapped difference test of network N4.

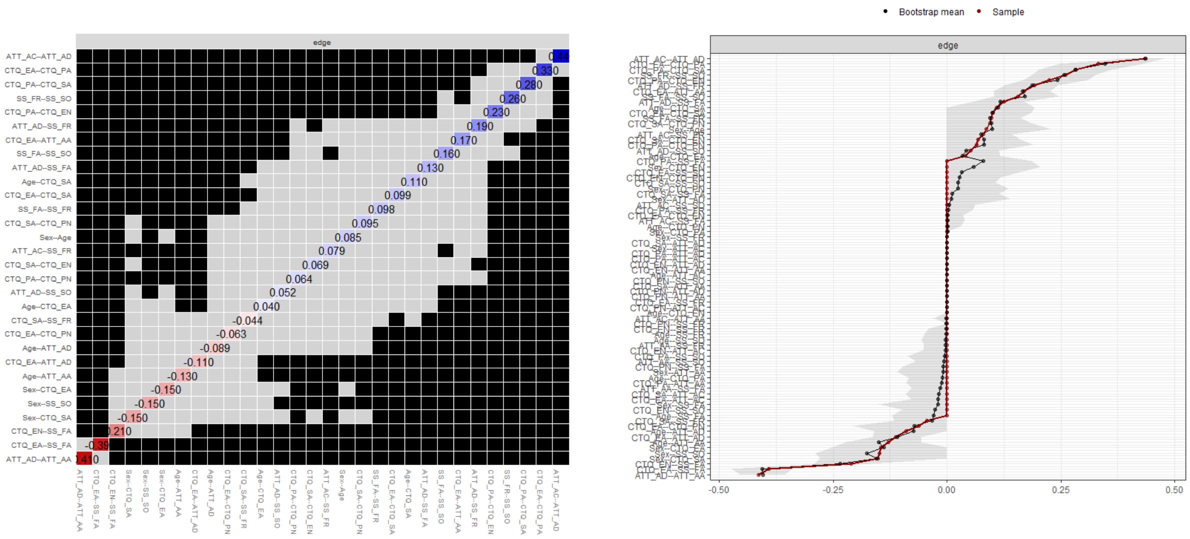

Fig. S14. Edge Weights and bootstrapped difference test of network N4.
